# Translation and cross-cultural adaptation of seventeen widely-used assessment instruments for child and adolescent mental health in Greece

**DOI:** 10.1186/s41687-024-00693-0

**Published:** 2024-02-12

**Authors:** Vasiliki Eirini Karagiorga, Julia Luiza Schafer, Lauro Estivalete Marchionatti, Arthur Caye, Aspasia Serdari, Konstantinos Kotsis, Maria Basta, Panagiota Balikou, Efstathia Kapsimalli, Andromachi Mitropoulou, Nikanthi Klavdianou, Domna Zeleni, Sotiria Mitroulaki, Anna Botzaki, Giorgos Gerostergios, Giorgos Samiotakis, André Simioni, Katholiki Georgiades, Giovanni Abrahão Salum, Anastasia Koumoula

**Affiliations:** 1 Child and Adolescent Mental Health Initiative (CAMHI), Stavros Niarchos Foundation and Child Mind Institute, 101 East 56th Street, New York, New York 10022 USA; 2 Child Mind Institute, New York, USA; 3 Department of Psychiatry, Universidade Federal do Rio Grande do Sul (UFRGS), Porto Alegre, Brazil; 4 Department of Child and Adolescent Psychiatry, Medical School, Democritus University of Thrace, Alexandroupolis, Greece; 5 Department of Psychiatry, Faculty of Medicine, School of Health Sciences, University of Ioannina, Ioannina, Greece; 6 Department of Psychiatry, University Hospital of Heraklion, Crete, Greece; 7 Department of Child and Adolescent Psychiatry, University Hospital of Heraklion, Crete, Greece; 8 Department of Psychiatry and Behavioural Neurosciences and Offord Centre for Child Studies, McMaster University, Hamilton, Canada

**Keywords:** Instruments, Measurement, Psychometrics, Scales, Mental health

## Abstract

**Background:**

In the context of Greece, many instruments measuring constructs pertinent to child and adolescent mental health lacked a locally-validated, freely-available version. As part of a nationwide survey, we translated and cross-culturally adapted a collection of seventeen brief, largely-employed assessment tools that can be used at scale.

**Methods:**

This study is part of the Child and Adolescent Mental Health Initiative in Greece (CAMHI), a capacity-building program focusing on enhancing mental health care for children and adolescents living in Greece. We conducted a nationwide survey assessing mental health symptoms, parenting practices, service availability and quality, mental health literacy and stigma, and professional practices within the country. As part of this process, we selected outcomes and instruments after consulting the International Consortium for Health Outcomes Measurement (ICHOM) and the COnsensus-based Standards for the selection of health Measurement INstruments (COSMIN). From our selection, we identified 17 instruments that did not have a Greek-validated version available for use. These instruments were translated and cross-culturally adapted following a structured procedure, including independent back-and-forth translations, synthesis of versions, expert revision, and pilot testing. Some instruments were slightly modified to meet CAMHI survey purposes.

**Results:**

A cross-culturally adapted version in Greek was made available for the following instruments: Pediatric Symptoms Checklist (PSC); Deliberate Self Harm Inventory (DSH) (modified); Child and Adolescent Trauma Screen-2 (CATS-2); ABCD Screen Use (modified); Swanson, Nolan, and Pelham-IV (SNAP-IV); Parent Behavior Inventory (PBI); Mental Health Literacy Scale (MHLS)—(modified); Australian Mental Health Vignettes; Reported and Intended Behavior Scale (RIBS); Barriers to Access to Care (BACE) (modified); Experience of Service Questionnaire (ESQ) (modified); and Multitheoretical List of Therapeutic Interventions (MULTI-30) (modified).

**Conclusion:**

A collection of these widely-used assessment tools is now adapted for the local context and freely accessible at [https://osf.io/crz6h/]. Researchers and health professionals in Greece can utilize this resource to screen, evaluate, and monitor various constructs related to mental health in accordance with the most effective practices.

**Supplementary Information:**

The online version contains supplementary material available at 10.1186/s41687-024-00693-0.

## Background

Assessment instruments are essential for objective and reliable evaluation of outcomes in mental health services and research [1]. These tools are designed to measure mental health conditions, allowing to determine the prevalence of disorders at the research level and provide means for clinically screening, diagnosing, and classifying the severity of conditions. Moreover, these instruments play a pivotal role in monitoring changes over time, such as assessing response to treatment protocols, which is a cornerstone of the measurement-based care approach currently considered a best practice [2]. Despite their utility, a significant proportion of these instruments are originally developed in English and their assessed mental health concepts cannot be assumed to be stable cross-culturally [3, 4]. As a result, their accessibility and applicability in non-English speaking regions may be limited thus requiring a validated and rigorous process of cultural adaptation [5].

In the context of Greece, a recent systematic review compiled instruments for child and adolescent mental health that were either developed in or validated for the Greek language [4]. Data on 261 instruments were analyzed from 223 studies, cataloging the available tools according to conditions, informants, and validated properties. The literature on assessment instruments reveals a variety of tools available for evaluating mental health constructs, specifically for domains such as neurodevelopment or broadband constructs of mental disorders. However, a significant portion of these instruments lack appropriate cross-cultural adaptation, as the majority of instruments were translated from their original English version using a simple back-and-forth procedure. Furthermore, the extent and availability of instruments are limited, as some are behind fee-for-use paywalls. Finally, there was a scarcity of measurement scales in the Greek language for key domains such as substance use, psychotic disorders, or disruptive behavior, adding barriers to adequate assessment of these conditions in the country.

The Child and Adolescent Mental Health Initiative is a capacity-building program to strengthen the mental health provision for children and adolescents in Greece. As part of the program, our team conducted a nationwide survey on the current state and needs within the country, covering mental health symptoms, parenting behaviors, literacy and stigma, service use and access, and professional practices [6]. Upon consulting available literature, we identified an absence of locally-adapted instruments to measure these constructs, underscoring a significant gap in assessment tools for child and adolescent mental health in the country. To address this shortfall, we present the translation and cross-cultural adaptation process of 17 widely-employed mental health instruments that are now newly and freely available for the Greek context.

## Methods

This work is part of a nationwide survey on mental health needs/services for children and adolescents in Greece [6]. Within the survey, we consulted the International Consortium for Health Outcomes Measurement (ICHOM)—an organization focusing on defining and standardizing the measurement of health outcomes that matter to patients—on recommendations for selecting outcomes related to mental health symptoms, parenting, mental health literacy and stigma, service use and access, and professional practices [7, 8]. Following the COnsensus-based Standards for the selection of health Measurement INstruments (COSMIN), we selected instruments providing free and reliable measures on such outcomes, prioritizing brief questionnaires to facilitate application in reduced time [9]. We searched the literature for locally-validated versions of these instruments, and if a Greek version was not available, we performed a cross-cultural adaptation procedure from an English version. A collection of 17 instruments were then translated and adapted to Greek (see Table 1 for a detailed description, including references for consulting the psychometric properties of the original instruments such as content, criterion and construct validity, factor structure, and internal consistency).

**Table 1:** Description of cross-culturally adapted instruments

Domain	Instrument	Informant	Number of items (final version)	Measured construct
Mental health symptoms	Pediatric symptoms checklist (PSC-Y) [10] + questions on daily life impact	Child/adolescent	35 + 5	Psychosocial functioning
	Pediatric symptoms checklist (PSC-C) [10] + questions on daily life impact	Caregiver		
	Deliberate self harm inventory (DSHI-9) modified short version (modified to use in the CAHMI) [11]	Child/adolescent	11	Non-suicidal self injury
	Child and adolescent trauma screen-2 (CATS2-C) [12]	Caregiver	16–41	Traumatic exposure and post-traumatic stress disorder symptoms
	Child and adolescent trauma screen-2 (CATS2-Y) [12]	Child/adolescent		
	UCSD ABCD screen use-C (modified to use in the CAHMI) [13]	Caregiver	6	Time spent in screen use
	UCSD ABCD screen use-Y (modified to use in the CAHMI) [14]	Child/adolescent	11	
	SNAP-IV (Swanson, Nolan, and Pelham) [15]	Caregiver	26	Hyperactivity, inattention, impulsivity, and oppositional behavior
Parenting	Parent behavior inventory (PBI) [16]	Caregiver	20	Parenting behavior as of two subscales: supportive/engaged and hostile/coercitive
Mental health literacy and stigma	Mental health literacy scale (MHLS) (modified to use in the CAHMI) [17]	Caregiver and general population	28	Literacy and attitudes on mental health disorders
	Australian mental health vignettes [18–20]	Child/adolescent	2 vignettes 26 questions	Mental health vignettes depicting common conditions to elicit audience attitudes
	The reported and intended behavior scale (RIBS) [21]	Caregiver and general population	8	Previous social contact and intentions towards people facing mental health issues
Service use and access	Barriers to access to care evaluation (BACE) (modified to use in the CAHMI) [22]	Caregiver	30	Barriers to care experienced by people who use or have used mental health services
	Experience of service questionnaire (ESQ-C) (modified to use in the CAHMI) [23]	Caregiver	12	Satisfaction of use with mental health services
	Experience of service questionnaire (ESQ-Y 12–17 years) (modified to use in the CAHMI) [23]	Adolescent		
	Experience of service questionnaire (ESQ-Y 9–11 years) (modified to use in the CAHMI) [23]	Child		
Professional practices	The multitheoretical list of therapeutic interventions (MULTI-30) (modified to use in the CAHMI) [24]	Healthcare professionals	30	Frequency of use of diverse mental health skills and techniques

*Notes* *two mental health vignettes. Some instruments were modified to use in the Child and Adolescent Mental Health Initiative, as detailed in Table 2 and Supplementary Table 1

**Fig. 1:**
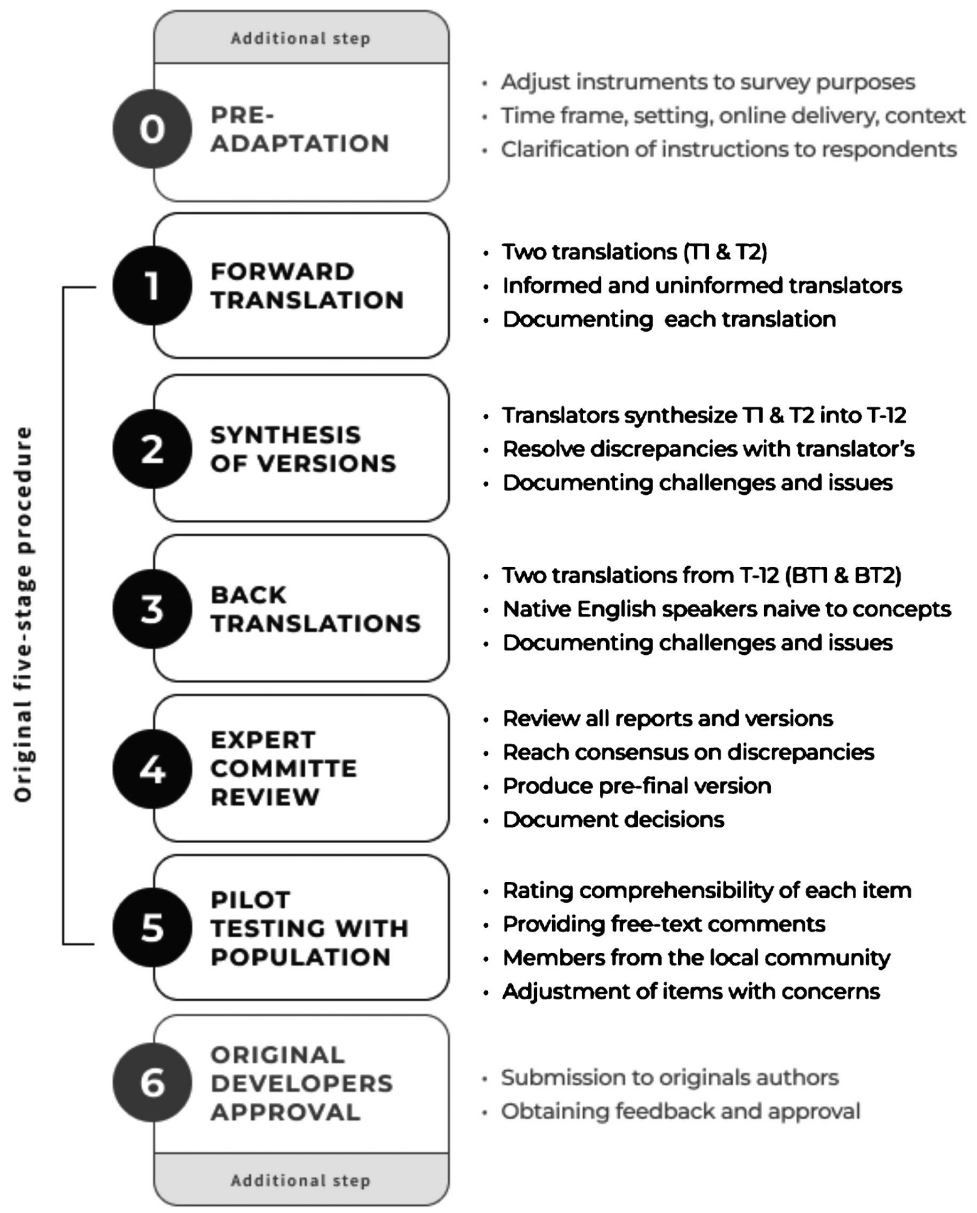
Cross-cultural adaptation procedure

For performing cross-cultural and linguistic adaptation, we followed a well-established five-stage procedure (see Fig. 1) [25]. This is a vastly-replicated method that was chosen for its rigorous and structured approach, including two-way-translation by independent translators, expert equivalence verification, and piloting with the targeted population. We added two further stages. As an initial step, we pre-adapted some instruments to reflect research purposes of the survey (such as timeframe, raters, setting, and other contextual factors), sometimes assembling a working version of the questionnaires from their existing variations. As a final step, we returned the cross-culturally adapted versions to the original authors for their comments and final approval.

For each instrument, the steps of the translation and cross-cultural adaptation process were registered in an item-by-item manner. This documentation can be consulted as a supplementary material in [https://osf.io/crz6h/].

### Stage 0: Pre-adaptation of the original instruments for the nationwide survey

Before translation, some instruments were pre-adapted to meet the survey purposes and to be tailored to the characteristics of the audience, without altering their core meaning or structure. These adaptations included adjusting questions to reflect the timeframe of evaluation, as well as to match the right informant for the scale. We also made pertinent clarifications on the instructions for respondents and adjusted the instruments to online use. For some questionnaires, we assembled a version of the instruments by selecting sections of different available forms of the tool. Instruments that were included were freely accessible for use or permission was sought from their original developers. The King’s College London granted special consent for adaptation of the Barriers to Access to Care Evaluation (BACE) for assessing barriers to care for children and adolescents as reported by caregivers.

### Stage 1: Initial Greek translations

Two forward translations were independently performed by two bilingual Greek native speakers with different professional backgrounds: The first translator was a child and adolescent psychiatrist with expertise in the concepts being examined in the questionnaires. The second translator was naive to mental health concepts and belonged to the certified staff of an official Greek translation company (involving translators who are unfamiliar to the specific technical background is a crucial step in attaining a conceptual understanding of items for the general population). Each translator received an excel worksheet in a standard format to translate the instruments and record any inconsistencies or considerations. The translators produced written reports to support their translation, including challenging phrases or uncertainties and the rationale for their choices.

### Stage 2: Synthesis of the Greek translations

The two forward translators synthesized the results of the two individual Greek versions. Working from the two translations and corresponding reports, they reached a consensus on any discrepancies. This generated an unified translation, which was accompanied by written observations on the challenges that were encountered as well as choices taken.

### Stage 3: Back translation to English

As part of a validity checking step, independent back translations from Greek to English were then produced by two bilingual English native speakers. These translators had no medical or psychological training and were unaware of the concepts being explored. One was an administrative member of the CAMHI, whilst the other was a certified translator based in the USA. Written observations were documented for challenging points of translation.

### Stage 4: Expert committee producing a pre-final Greek version

The expert committees were composed of four to six Greek mental health experts (child and adolescent psychiatrists, psychologists, and special educators), all members of the CAMHI team, including the first forward translator. The role of the committee was to consolidate all versions for each instrument and develop a pre final version for pilot testing. The committee reviewed the original questionnaire, as well as all forward and back translations and their documentation, reaching a consensus on any discrepancies. Crucial decisions made by the committee were reported in the documentation. Decisions were aimed at achieving semantic, idiomatic, experiential, and conceptual equivalence between the original and the translated versions [25]. Semantic equivalence refers to ensuring that the translated text carries the same meaning as the original text. Idiomatic equivalence pertains to the translation of expressions or phrases that have a non-literal meaning, including colloquialisms. Experiential equivalence ensures that the translated text reflects the daily life experiences of the target culture. Conceptual equivalence deals with the translation of concepts that may carry different underlying interpretations or principles in different cultures.

### Stage 5: Pilot testing with the population

The preliminary Greek version of each instrument was pre-tested with community samples to evaluate the level of understandability per question and response item. Convenience sampling was performed across different regions in the country where the CAHMI hubs are located (Athens, Alexandroupoli, Ioannina, Crete) aimed at recruiting members from targeted age groups (children, adolescents, caregivers of children, caregivers of adolescents) and health professionals. Participants were approached in-person to rate both language and conceptual comprehension on a 5-point Likert scale (*I didn’t understand anything, I understood a little, I understood most of it, I understood a lot, I totally understood*), commenting on items with a low rating. Free-text feedback was also incentivized to provide insights into the associated meanings of sentences and words, especially in items that testers judged problematic or potentially ambiguous. The responses were collected electronically using the software “kobotoolbox” [26], which also provides automatic reports and computerized calculations. Responses were then examined by the CAMHI’s expert team and items rated ‘*I didn’t understand anything*’ or ‘*I understood a little*’ or with concerning comments by most participants were reformulated.

Approval for the pilot testing was granted by the Research Ethics Committee of the Democritus University of Thrace [approval number: ∆ΠΘ/ΕΗ∆Ε/42772/307]. Electronic consent forms were signed by participants and data was handled unidentified.

### Stage 6: Submission of final version to the original authors

The final Greek version of each instrument was sent to the original developers, alongside detailed reports on the processes observed at each translation stage in Greek. This stage was aimed at obtaining their final approval as well as relevant inputs.

## Results

A cross-culturally adapted version in Greek was made available to a collection of 17 broadly-employed instruments for mental health related-outcomes, namely: Pediatric Symptoms Checklist (PSC) plus impact questions—caregiver and child/adolescent versions [10]; Deliberate Self Harm Inventory (DSH) (modified to use in the CAHMI) [11]; Child and Adolescent Trauma Screen-2 (CATS-2)—caregiver and child/adolescent versions [12]; ABCD Screen Use—caregiver and child/adolescent versions (modified to use in the CAHMI) [13]; Swanson, Nolan, and Pelham-IV (SNAP-IV) [15]; Parent Behavior Inventory (PBI) [16]; Mental Health Literacy Scale (MHLS) (modified to CAMHI survey purposes) [17]; Australian Mental Health Vignettes [18–20]; Reported and Intended Behavior Scale (RIBS) [21]; Barriers to Access to Care (BACE) (modified to use in the CAHMI) [22]; Experience of Service Questionnaire (ESQ) (modified to use in the CAHMI)—caregiver, adolescent, and child versions [23]; and The Multitheoretical List of Therapeutic Interventions (MULTI-30) (modified to use in the CAHMI) [24]. The final version of the instruments are available at [https://osf.io/crz6h/], alongside the documentation of the cross-adaptation procedure. Below, we describe relevant details of each stage of the process.

### Stage 0: Pre-adaptation of the original instruments for the nationwide survey

A description of the preadaptation of instruments is available in Table 2 (see Supplementary Table 1 for detailed rationale and modifications; see Table 1 for the number of items in the final version of the instrument). Mainly, such modifications related to instructions’ clarity or brevity, to match online survey delivery, to adapt time frame of assessment (e.g., to reflect satisfaction on previous health service use, as opposed to a present visit), or to changes to the informant of the instrument (e.g., service use scales were originally rated by adult participants on their own use of services, thus the questions were altered to clarify that they were rating the services used by their child/adolescent). Two instruments were assembled by selecting appropriate items or sections from different existing versions. An additional section on daily impact of symptoms was added to the Pediatric Symptoms Checklist, similar to existing behavior screening tools [27–29].

**Table 2:** Pre-adaptation of instruments

**Pediatric symptoms checklist** and daily impact questions: youth (PSC-Y) and caregiver (PSC-C) versions	Addition of five questions assessing the daily life impact (as in widely used behavioral screening questionnaires) [27–29]
**Deliberate self harm inventory (DSHI-9)** modified short version.	Assemblage of elements from two scales adapted for adolescent use [30, 31]
**Child and adolescent trauma screen-2:** caregiver (CATS2-C) and youth (CATS2-Y) version	Adaptation of questions on work to reflect local child/adolescent contexts Instructions were simplified
**UCSD ABCD screen:** caregiver (Use-C) and youth (USE-Y) versions	Assembled from the ABCD questionnaires reflecting activities of interest within the Greek context [13, 14, 32–36] Removed items concerning habits during the COVID-19 pandemic
**Mental health literacy scale (MHLS)**	Omission of 6 items on knowledge of available assistance and engagement in help-seeking behavior
**Australian mental health vignettes**	Adaptations of the age of the portrayed children to suit both children and adolescents. Addition of social workers on the list of professionals listed as options to mark probability of help-seeking behavior due to the cultural relevance of this speciality in Greece
**The reported and intended behavior scale (RIBS)**	Shortening of instructions and adaptation to online survey
**Barriers to access to care evaluation** (BACE)* * adapted with the consent of King’s College London	Modified to a caregiver report on child/adolescent barriers to care (originally adult self-report) Instructions shortened Adaptation of questions on work to reflect child/adolescent contexts
**Experience of service questionnaire:** caregiver (ESQ-C)^:^, adolescent (ESQ-Y—12–17 years) and child (ESQ-Y—9–11 years) versions	Modified to reflect past use of services (versus ongoing use) Exclusion of free-text sections on constructive feedback
**The multitheoretical list of therapeutic interventions** (MULTI-30)	Adaptation to a self-report version of practicing therapists to report their own interventions targeting only adolescents and young adults

### Stages 1–4: Translation, back-translation, expert committee

Forward- and back-translation procedures were performed and the synthesis of versions occurred without relevant discrepancies. The expert committee determined the pre final version of each instrument. A few noteworthy modifications resulted from these stages. The Barriers to Access to Care Evaluation (BACE) had the original English phrase “the child/adolescent I am responsible for” replaced with the terms “my child/adolescent”, removing the resulting incomprehensible prolixity in the Greek translation. The Experience of Service Questionnaire (ESQ-Y 12–17 years) adolescent version had the 4-point Likert scale rephrased to address the resulting language complexity in Greek translation, which would be inappropriate for this age range in the local culture. In the Swanson, Nolan, and Pelham IV questionnaire (SNAP-IV), the term child/adolescent in the introductory instructions text was simplified to include only the word child in Greek, which is a term that embraces all youth age-ranges in local culture.

### Stage 5: Initial testing

Table 3 provides an overview of the piloting of instruments, and a more detailed description is available in Supplementary Table 2. Most items were awarded a score of 4 (“*I understood a lot*”) or 5 (“*I totally understood*”) by the majority of participants. A small number of items that scored below 3 (“*I didn’t understand anything*” or “*I understood a little*”), or those that received text feedback indicating potential issues, were referred back to the committee for final modifications. Instructions and response options were also susceptible to revisions, which were implemented in a few cases. Most instruments remained unmodified after pilot testing; a few tools underwent slight alterations in wording for specific items that did not affect their associated meanings. Considering that, the reformulated instruments were not subjected to a retest.

**Table 3:** Pilot testing

Instrument	Sample	Modified items
**Pediatric symptoms checklist (PSC-Y) + daily life impact questions** Rating: children and adolescents	10 participants 6–17 years old (mean 13.20) 60% female	No further modifications needed
**Pediatric symptoms checklist (PSC-Y) + daily life impact questions** Rating: caregivers	5 caregivers responsible for one child/adolescent each Children age: 6–13 years old (mean 8.80) Children gender: 40% female	No further modifications needed
**Swanson, Nolan, and Pelham IV questionnaire (SNAP-IV)** Rating: caregivers	6 caregivers responsible for one child/adolescent each Children age: 6–15 years old (mean 12.00) Children gender: 66.67% female	No further modifications needed
**Deliberate self harm inventory (DSHI-9)** Rating: adolescents	6 participants 15–17 years old (mean 16.16) 83.33% female	No further modifications needed
**Child and adolescent trauma screen-2 (CATS2-Y)** Rating: children and adolescents	9 participants 9–17 years old (mean 14.11) 77.78% female	Wording adjustments of 8 items and instructions
**Child and adolescent trauma screen-2 (CATS2-C)** Rating: caregivers	8 caregivers responsible for one child/adolescent each Children age: 2–16 years old (mean 9.50) Children gender: 25% female	Wording adjustments of 28 Greek items (mainly to include all gender pronouns)
**UCSD ABCD screen use- youth** Rating: adolescents	5 participants 12–16 years old (mean 14.60) 40% female	No further modifications needed
**UCSD ABCD screen use- caregiver** Rating: caregivers	7 caregivers responsible for one child/adolescent each Children age: 6–15 years old (mean 12.14) Children gender: 71.43% female	No further modifications needed
**Parent behavior inventory (PBI)** Rating: caregivers	6 caregivers responsible for one child/adolescent each Children age: 6–15 years old (mean 7.83) Children gender: 33.33% female	Reformulating the 6-point Likert scale of all items
**Mental health literacy scale (MHLS)** Rating: caregivers and teachers	1 teacher 6 caregivers responsible for one child/adolescent each Children age: 3–15 years old (mean 8.57) Children gender: 57,14% female	No further modifications needed
**Australian mental health vignettes** Rating: child and adolescents	5 participants 11–17 years old (mean 14.17) 40% female	Wording adjustments of 4 items
**The reported and intended behavior scale (RIBS)** Rating: caregivers, teachers, and health professionals	6 caregivers responsible for one child/adolescent each Children age: 3–15 years old (mean 9,67) Children gender: 50% female	No further modifications needed
**Barriers to access to care evaluation (BACE)** Rating: caregivers	8 caregivers responsible for one child/adolescent each Children age: 5–15 years old (mean 8.75) Children gender: 62,5% female	Wording adjustments of 2 items
**Experience of service questionnaire (ESQ-C)** Rating: caregivers	6 caregivers responsible for one child/adolescent each Children age: 5–15 years old (mean 10.33) Children gender: 66,66% female	Wording adjustments of the instructions text
**Experience of service questionnaire (ESQ-Y 9-11),** Rating: child	5 participants 9–11 years old (mean 9.80) 40% female	Wording adjustments of 1 Greek item and the instructions text
**Experience of service questionnaire (ESQ-Y 12-17)** Rating: adolescent	7 participants 12–17 years old (mean 15.57) 100% female	No further modifications needed
**The multitheoretical list of therapeutic interventions (MULTI-30)** Rating: healthcare professionals	7 adult participants 27–47 years old (mean 39.86) 100% female	Wording adjustments of 4 items

### Stage 6: Submission of final version to the original authors

Original authors inspected and approved the final version of the instruments. No further modifications resulted from this stage.

## Discussion

A collection of seventeen internationally-recognized and widely-used instruments not initially available in the Greek language are now cross-culturally translated and adapted to Greek, encompassing tools that measure relevant constructs for child and adolescent mental health care such as mental health symptoms, screen use, substance use, self-harm, parenting behavior, literacy and stigma, access to care, satisfaction with services, and professional practices (see Table 1 for a full description of each instrument). These instruments can now be freely accessible at [https://osf.io/crz6h/], providing tools for researchers, clinicians, and policymakers in the country and allowing application with local samples for future psychometric validation. To the best of our knowledge, this is the first task force to compile a whole set of instruments for child and adolescent mental health outcomes for a particular country.

Having regionally-adapted, openly-accessible scientific tools is crucial to implement evidence-based practices within local contexts. Yet, the majority of tools are developed and made available in English, leading to obstacles for culturally appropriate measurement. A recent examination of the literature on child and adolescent mental health in Greece underscored this issue, highlighting that most assessment instruments were primarily developed in English and lacked rigorous validation for use in the Greek language [4]. It also revealed that many instruments were protected by paywalls. Licensed questionnaires may incur fees to cover research costs, and while some licenses offer free access for clinical and academic purposes, pay-for-use distribution may impose barriers to real-world practice. Following principles of dissemination and implementation science, evidence-based tools should be unrestricted to use and readily accessible to practitioners, researchers and policy makers to maximize their impact [37, 38]. The new set of regionally-adapted tools made available by this initiative can enable professionals to adhere to best practices and conduct assessments aligned with evidence-based practices, potentially enhancing local scientific practices. As in Greece, many non-English speaking countries face a shortage of instruments for child and adolescent mental health in their spoken languages, particularly in low-to-middle income nations [39–41]. Similar task forces could contribute to delivering resources in underserved settings, advancing evidence-based mental health assessment globally.

Our cross-cultural adaptation employed a structured five-stage procedure that has been vastly-replicated in the literature [25]. This process was carried out by a team of translators and experts, involving comprehensive stages such as two independent forward and backward translations, committee judgment for linguistic equivalence, and pilot testing with samples of the targeted population. A limitation of this procedure is not estimating experts’ agreement on the content validity of each item. Nevertheless, a panel of specialists presented few to none discrepancies throughout the process, and a quantification of comprehensibility was derived from structured questionnaires applied in the pilot testing with the target populations. In accordance with the principles of the open-science framework, the translation process was thoroughly documented and reported as supporting material [https://osf.io/crz6h/], enhancing credibility and reproducibility [42, 43]. Nevertheless, a notable limitation was the restricted sample size achieved during recruitment. Due to the extensive number of instruments that needed to be rated, we could only engage a medium of 6.29 participants per tool. This is below the number of thirty individuals considered ideal by the original procedure [25]. We also conducted minor adjustments in the instruments for suiting the survey purposes. Although these were confined to settings and timeframes without modifying the conceptual foundation of the scales, they are arguably an alteration from original documents. Beyond the scope of the present work, the psychometric validation will be performed with the nationwide survey sample.

## Conclusion

The present initiative has undertaken the cross-cultural adaptation and translation to the Greek language of 17 internationally-recognized instruments related to mental health constructs, making them freely available for researchers, clinicians, and policymakers in the country. This collection addresses previous gaps on the availability of locally-adapted instruments in Greece and provides a means to assess critical domains in the country, which is a vital step for implementing evidence-based practices within local contexts. Upcoming research should test the instruments in large samples in the Greek population for validating their psychometric properties.

### Electronic supplementary material

Below is the link to the electronic supplementary material.

Supplementary Material 1

## Data Availability

The datasets generated during the current study are available in the Open Science Framework repository [https://osf.io/crz6h/].

## Abbreviations

BACE: Barriers to access to care
COSMIN: COnsensus-based standards for the selection of health measurement instruments
CAMHI: Child and adolescent mental health initiative
CATS-2: Child and adolescent trauma screen-2
DSH: Deliberate self harm inventory
ESQ: Experience of service questionnaire
ICHOM: International consortium for health outcomes measurement
MHLS: Mental health literacy scale
MULTI-30: Multitheoretical list of therapeutic interventions
PBI: Parent behavior inventory
PSC: Pediatric symptoms checklist
RIBS: Reported and intended behavior scale
SNAP-IV: Swanson, Nolan, and Pelham-IV

## References

[1] Deighton J, Croudace T, Fonagy P (2014). Measuring mental health and wellbeing outcomes for children and adolescents to inform practice and policy: a review of child self-report measures. Child Adolesc Psychiatr Ment Health.

[2] Parikh A, Fristad MA, Axelson D, Krishna R (2020). Evidence base for measurement-based care in child and adolescent psychiatry. Child Adolesc Psychiatr Clin N Am.

[3] Ali G-C, Ryan G, De Silva MJ (2016). Validated screening tools for common mental disorders in low and middle income countries: a systematic review. PLoS One.

[4] Koumoula A, Marchionatti LE, Caye A et al (2023) The science of child and adolescent mental health in Greece: a nationwide systematic review. Eur Child Adolesc Psychiatry. 10.1007/s00787-023-02213-9 10.1007/s00787-023-02213-9 PMC11564383 37179505

[5] Gilmoor AR, Adithy A, Regeer B (2019). The cross-cultural validity of post-traumatic stress disorder and post-traumatic stress symptoms in the Indian context: a systematic search and review. Front Psychiatry.

[6] Koumoula A, Marchionatti LE, Karagiorga VE et al (2023) Understanding priorities and needs for child and adolescent mental health in Greece from multiple informants: an open resource. medRxiv 10.1007/s00787-024-02400-2 PMC11564210 38558204

[7] Terwee CB, Zuidgeest M, Vonkeman HE (2021). Common patient-reported outcomes across ICHOM Standard Sets: the potential contribution of PROMIS®. BMC Med Inform Decis Mak.

[8] International Consortium for Health Outcomes Measurement (ICHOM) (2018) International consortium for health outcomes measurement (ICHOM). In: ICHOM - value based healthcare, improving patient outcomes. https://www.ichom.org/. Accessed 2 Jun 2023

[9] Prinsen CAC, Mokkink LB, Bouter LM (2018). COSMIN guideline for systematic reviews of patient-reported outcome measures. Qual Life Res.

[10] Jellinek MS, Murphy JM, Robinson J (1988). Pediatric symptom checklist: screening school-age children for psychosocial dysfunction. J Pediatr.

[11] Bjärehed J, Lundh L-G (2008). Deliberate self-harm in 14-year-old adolescents: how frequent is it, and how is it associated with psychopathology, relationship variables, and styles of emotional regulation?. Cogn Behav Ther.

[12] Sachser C, Berliner L, Risch E (2022). The child and adolescent trauma screen 2 (CATS-2) - validation of an instrument to measure DSM-5 and ICD-11 PTSD and complex PTSD in children and adolescents. Eur J Psychotraumatol.

[13] Bagot KS, Tomko RL, Marshall AT (2022). Youth screen use in the ABCD® study. Dev Cogn Neurosci.

[14] Nagata JM, Cortez CA, Cattle CJ (2022). Screen time use among US adolescents during the COVID-19 pandemic: findings from the adolescent brain cognitive development (ABCD) study. JAMA Pediatr.

[15] Bussing R, Fernandez M, Harwood M (2008). Parent and teacher SNAP-IV ratings of attention deficit hyperactivity disorder symptoms: psychometric properties and normative ratings from a school district sample. Assessment.

[16] Lovejoy MC, Weis R, O’Hare E, Rubin EC (1999). Development and initial validation of the parent behavior inventory. Psychol Assess.

[17] O’Connor M, Casey L (2015). The mental health literacy scale (MHLS): a new scale-based measure of mental health literacy. Psychiatry Res.

[18] Jorm AF, Wright A, Morgan AJ (2007). Beliefs about appropriate first aid for young people with mental disorders: findings from an Australian national survey of youth and parents. Early Interv Psychiatry.

[19] Reavley NJ, Jorm AF (2011). Recognition of mental disorders and beliefs about treatment and outcome: findings from an Australian national survey of mental health literacy and stigma. Aust N Z J Psychiatry.

[20] Jorm AF, Wright A (2008). Influences on young people’s stigmatising attitudes towards peers with mental disorders: national survey of young Australians and their parents. Br J Psychiatry.

[21] Evans-Lacko S, Rose D, Little K (2011). Development and psychometric properties of the reported and intended behaviour scale (RIBS): a stigma-related behaviour measure. Epidemiol Psychiatr Sci.

[22] Clement S, Brohan E, Jeffery D (2012). Development and psychometric properties the barriers to access to care evaluation scale (BACE) related to people with mental ill health. BMC Psychiatry.

[23] Brown A, Ford T, Deighton J, Wolpert M (2014). Satisfaction in child and adolescent mental health services: translating users’ feedback into measurement. Adm Policy Ment Health.

[24] Solomonov N, McCarthy KS, Gorman BS, Barber JP (2019). The multitheoretical list of therapeutic interventions - 30 items (MULTI-30). Psychother Res.

[25] Beaton DE, Bombardier C, Guillemin F, Ferraz MB (2000). Guidelines for the process of cross-cultural adaptation of self-report measures. Spine.

[26] KoboToolbox. In: KoboToolbox. https://www.kobotoolbox.org/about-us/. Accessed 28 May 2023

[27] Salum GA, Gadelha A, Pan PM (2015). High risk cohort study for psychiatric disorders in childhood: rationale, design, methods and preliminary results. Int J Methods Psychiatr Res.

[28] Goodman R, Ford T, Simmons H (2000). Using the strengths and difficulties questionnaire (SDQ) to screen for child psychiatric disorders in a community sample. Br J Psychiatry.

[29] Goodman A, Heiervang E, Collishaw S, Goodman R (2011). The “DAWBA bands” as an ordered-categorical measure of child mental health: description and validation in British and Norwegian samples. Soc Psychiatry Psychiatr Epidemiol.

[30] Lundh L-G, Karim J, Quilisch E (2007). Deliberate self-harm in 15-year-old adolescents: a pilot study with a modified version of the deliberate self-harm inventory. Scand J Psychol.

[31] Gratz KL (2001) Measurement of deliberate self-harm: preliminary data on the deliberate self-harm inventory1. http://www.selfinjury.bctr.cornell.edu/perch/resources/dshi.pdf. Accessed 9 Jun 2023

[32] National Institute for Mental Health (2020) ABCD parent screen time survey (STQ).

[33] Hardy LL, Booth ML, Okely AD (2007). The reliability of the adolescent sedentary activity questionnaire (ASAQ). Prev Med.

[34] RTI International (2007) Sitting-sedentary behavior. In: PhenX Toolkit: Protocols. https://www.phenxtoolkit.org/protocols/view/150602?origin=search. Accessed 9 Jun 2023

[35] National Institute for Mental Health (2021) ABCD sports activities read/music – youth. In: NIMH Data Archive. https://nda.nih.gov/data_structure.html?short_name=sports_activ_read_music01. Accessed 9 Jun 2023

[36] National Institute for Mental Health (2021) ABCD youth screen time survey (STQ). In: NIMH Data Archive. https://nda.nih.gov/data_structure.html?short_name=abcd_stq01. Accessed 9 Jun 2023

[37] Shelton RC, Lee M, Brotzman LE (2020). What is dissemination and implementation science?: an introduction and opportunities to advance behavioral medicine and public health globally. Int J Behav Med.

[38] Beidas RS, Stewart RE, Walsh L (2015). Free, brief, and validated: standardized instruments for low-resource mental health settings. Cogn Behav Pract.

[39] Acharya B, Basnet M, Rimal P (2017). Translating mental health diagnostic and symptom terminology to train health workers and engage patients in cross-cultural, non-English speaking populations. Int J Ment Health Syst.

[40] Freeman M (2022). The world mental health report: transforming mental health for all. World Psychiatry.

[41] Brohan E, Chowdhary N, Dua T et al (2023) The WHO mental health gap action programme for mental, neurological, and substance use conditions: the new and updated guideline recommendations. Lancet Psychiatry. 10.1016/S2215-0366(23)00370-X 10.1016/S2215-0366(23)00370-X 37980915

[42] Vicente-Saez R, Martinez-Fuentes C (2018). Open science now: a systematic literature review for an integrated definition. J Bus Res.

[43] Munafò MR, Nosek BA, Bishop DVM (2017). A manifesto for reproducible science. Nat Hum Behav.

